# Fructo-Oligosaccharide Alleviates Soybean-Induced Anaphylaxis in Piglets by Modulating Gut Microbes

**DOI:** 10.3389/fmicb.2018.02769

**Published:** 2018-11-20

**Authors:** Meinan Chang, Yuan Zhao, Guixin Qin, Xiaodong Zhang

**Affiliations:** ^1^Key Laboratory of Animal Production, Product Quality and Security, Ministry of Education, Jilin Provincial Key Laboratory of Animal Nutrition and Feed Science, College of Animal Science and Technology, Jilin Agricultural University, Changchun, China; ^2^Institute of Zoonosis, Department of Public Health, Jilin University, Changchun, China

**Keywords:** soybean antigen, fructooligosaccharides, gut microbes, immune response, piglets

## Abstract

Soybean-induced anaphylaxis poses a severe threat to the health of humans and animals. Some commensal bacteria, such as *Lactobacillus* and *Bifidobacteria*, can prevent and treat allergic diseases. Prebiotic oligosaccharides, a food/diet additive, can enhance health and performance via modulating gut microbes and immune responses. The purpose of this study was to examine whether fructo-oligosaccharides (FOS) could alleviate soybean-induced anaphylaxis by modulating gut microbes. Piglets (21 days of age) were sensitized with a diet containing 5% soybean and 30% peeled soybean meal. The treatment with 0.6% FOS started 1 day prior to sensitization and continued everyday thereafter. Blood was collected for measurements of immune indices. The DNA samples isolated from fresh intestinal contents of the middle jejunum (M-jejunum), posterior jejunum (P-jejunum), ileum, and cecum were used for gene sequencing based on 16S rRNA. Our results showed that there was an increase of glycinin-specific IgG, β-conglycinin-specific IgG, total serum IgG and IgE, and occurrence of diarrhea in piglets sensitized with soybean antigen. There was a decrease in interleukin 4 (IL-4) and IL-10 and an increase of interferon-γ (IFN-γ) in piglets with FOS treatment, compared with the piglets without FOS treatment. Improvement of intestinal microbes was indicated mostly by the increase of *Lactobacillus* and *Bifidobacteria* in M-jejunum and the decrease of *Proteobacteria* in P-jejunum and ileum. The correlation analysis indicated that FOS treatment decreased those closely related to the key species of gut microbes. These results suggest that FOS can alleviate soybean antigen-induced anaphylaxis, which is associated with increased *Lactobacillus* and *Bifidobacteria* in M-jejunum and declined *Proteobacteria* in P-jejunum and ileum of piglets.

## Introduction

Soybean is a species of legume and an ideal source of protein for humans and animals (Guo et al., 2007; Sun et al., 2008). However, many antinutritional factors are contained in soybeans, such as glycinin, β-conglycinin, agglutinin, which severely undermine its nutritional values (Huang et al., 2010; Zhao et al., 2011). Soybean-induced anaphylaxis in humans and young animals is attributed mainly to allergens such as glycinin and β-conglycinin (Van de Lagemaat et al., 2007). Patients with anaphylaxis often exhibit symptoms such as tremor, throat edema, rash, and acute asthma. Piglets with soybean-induced anaphylaxis can have symptoms such as diarrhea, dysbiosis of intestinal digestive system, and even death (Wang et al., 2014).

Previous studies have shown that traditional processing techniques such as heat treatment, high-pressure puffing, and fermentation (Tang and Ma, 2009; Zheng et al., 2017) can passivate allergens in soybeans. Recent studies demonstrated that the addition of exogenous active substances such as lipoic acid (Cho et al., 2004), plant extracts (Hao et al., 2010; Julie et al., 2010), probiotics (Ly et al., 2011), or prebiotics (Gourbeyre et al., 2013) can prevent and treat allergies effectively.

Commensal bacteria in the intestinal tract, such as *Lactobacillus* and *Bifidobacteria*, play a major role in maturation and homeostasis of the gut-associated immune system (Holt and Jones, 2000; Lanning et al., 2000). These microbes prevent and treat post-antibiotic diarrhea (Cruchet et al., 2003), allergic diseases (Shida et al., 2002; Fiocchi et al., 2012), and recurrence of inflammatory bowel disease (De Cruz et al., 2012).

Prebiotics are indigestible compounds that confer beneficial physiological effects on the host by modulating the composition and activity of microorganisms in the gut (Valcheva and Dieleman, 2016). Fructo-oligosaccharides (FOSs) are non-digestible oligosaccharides that are found naturally in a variety of fruits and vegetables such as artichokes, leeks, asparagus, potatoes, wheat, onions, bananas, and can stimulate the growth of *Bifidobacteria* and *Lactobacillus* in the colon of healthy individuals. It is reported that FOS can strengthen the immune function by regulating immunoglobulin, immune cells and their secreted inflammatory factors, as well as intestinal microbes (Delgado et al., 2012). Human studies indicate that galacto-oligosaccharides (GOS)/FOS can reduce significantly the plasma level of total IgE and IgG1, IgG2, and IgG3 in infants at risk for allergy (Van et al., 2010). In addition, Watanabe et al. (2008) showed that FOS can reduce 2, 4-dinitrofluorobenzene-induced contact hypersensitivity in mice by promoting proliferation of *Bifidobacteria*. Therefore, we hypothesized that dietary FOS can alleviate soybean antigen-induced anaphylaxis by modulating gut microbes. As pigs have similar genetic background and physiological characteristics as humans, they are used widely in research to study human health and diseases (Alizadeh et al., 2016). In this study, we evaluated the impact of FOS on anaphylaxis induced by soybean antigen protein in piglets by determining the expression of a series of inflammatory and immune factors (IgG, IgE, IFN-γ, IL-4, IL-6, IL-10, and TNF-α) and the gut microbes. We aimed to find an effective strategy to inhibit the sensitization of antigen protein and explore the regulatory mechanisms of “host–microbiome” interaction.

## Materials and Methods

### Animals

All piglets used in this experiment were approved by the Jilin Agricultural University Animal Care and Use Committee. Fifteen crossed (Yorkshine-Landrance-Duroc) barrows were weaned at 21 days of age and had an average weight of 6.81 ± 0.58 kg. The piglets were divided randomly into three groups of five each and housed in a livestock farmhouse located in Jilin Agricultural University, Changchun.

### Experimental Diets

The ingredients and nutrients of the diet are shown in Table 1. The diet was formulated to meet NRC (2012) requirements. Piglets were assigned to three groups: the control group, the allergy group (5% soybean and 30% peeled soybean meal), and the FOS group [5% soybean and 30% peeled soybean meal plus 0.6% FOS (Solarbio, Beijing, purity > 95%)]. The soybean was purchased from Jilin Agricultural University soybean experimental field. The control diet, devoid of soybean protein, contained bran (4.5%), whey powder (7.19%), casein (11.13%), fish meal (2.00%), and digester tankage (1.02%) as protein sources. However, the allergy diet contained peeled soybean meal (30%), soybean (5%), and bran (1.30%) as protein sources. Zeolite is inert and does not react chemically with food or body fluids. Therefore, it is commonly used as a vector for premix. In this study, the control diet and the allergy diet contained 0.8% zeolite, whereas the FOS diet contained 0.6% FOS instead of zeolite.

**Table 1:** Ingredient composition and nutrient levels of the diets.

Ingredient composition (%)	Dietary treatment	
	Control diet	Allergy diet
Peeled soybean meal		30.00
Soybean		5.00
Casein	11.13	
Zeolite	0.80	0.80
Corn	65.70	52.10
Bran	4.50	1.30
Limestone	1.42	1.89
Whey powder	7.19	0.00
Fish meal	2.00	0.00
Digested tankage	1.02	0.00
Salt	0.65	1.00
Sucrose	2.55	1.20
Vitamin mineral premix^∗^	0.85	1.00
Oil	1.10	2.95
Phosphate	0.58	2.00
Lysine	0.29	0.45
Threonine	0.12	0.19
Methionine	0.10	0.12
Total	100	100
**Chemical analysis**		
Net energy (kJ/kg)	10.24	10.24
Crude protein (%)	17.5	17.5
Lysine (%)	1.44	1.35
Methionine (%)	0.41	0.44
Threonine (%)	0.83	0.79
Arginine (%)	1.25	0.71
Leucine (%)	1.47	1.66
Isoleucine (%)	0.73	0.73
Calcium (%)	1.00	0.86
Phosphorus (%)	0.40	0.40
Natrium (%)	0.41	0.36
Chlorine (%)	0.62	0.53


*^∗^Premix provided per kilogram of complete diet: vitamin A, 45,000,000 IU; vitamin D3, 9,000,000 IU; vitamin E, 80,000 IU; vitamin K3, 5000 mg; vitamin B1, 8000 mg; vitamin B2, 20,000 mg; vitamin B6, 9000 mg; vitamin B12, 100 mg; nicotinamide, 100,000 mg; D-calpanate, 50000 mg; folic acid, 4000 mg; *D*-biotin, 500 mg; Cu, 320 mg; Fe, 175 mg; Zn, 125 mg; Mn, 55 mg; Se, 7.5 mg; and I, 20 mg.*

### Experimental Protocol and Sample Collection

After 7 days of adaptation, the piglets were treated at 28 days of age. The piglets in allergy group and FOS group were sensitized for the first 10 days by feeding allergy diet and subsequently boosted by feeding allergy diet on days 16–18 and on days 31–32. The control diet was given on all the other days. The piglets had free access to food and water and were weighed at the start and the end of the trial (day 0 and day 32) to calculate the average daily weight gain.

The blood samples were obtained from each piglet by vena cava puncture using 10 mL gel vacuum collective tubes on days 10, 25, and 32. These blood samples were taken at a 3 h interval after feeding the allergy diet, incubated for 30 min at 37°C, and then centrifuged for 20 min at 2,000 rpm/min. The supernatant was collected and stored at -80°C for measurements of immunoglobulin and cytokine levels.

At day 32, three piglets from each group were chosen randomly and euthanized. The contents of four intestinal segments including middle of jejunum (M-jejunum), posterior of jejunum (P-jejunum), ileum, and cecum were collected. These samples were snap freezing in liquid nitrogen and stored at -80°C until use.

### Analysis of Total Serum IgG, IgE, and Specific IgG Levels by ELISA

Total serum IgG and IgE antibody levels were determined by using swine ELISA kits (Lengtonbio, China) according to the manufacturer’s instructions. Glycinin-specific IgG and β-conglycinin-specific IgG antibody levels were determined by using an indirect ELISA as previously described by Sun et al. (2008). The 96-well microplates (Corning-Costar, United States) were coated with 10 μg/mL glycinin in carbonate buffer (pH = 9.6) at 4°C overnight. The plates were washed three times and blocked with bovine serum albumin blocking solution (2%). Swine serum (appropriately diluted) was added and incubated with biotinylated antipig IgG and horseradish peroxidase conjugates (Abcam, United Kingdom). After wash, the color reagent and stop solution were added and the optical density (OD) was measured at 450 nm. The IgG value was calculated based on the standard curve and dilution factors.

### Analysis of Cytokine Levels by ELISA

Concentrations of interleukin 4 (IL-4), IL-6, IL-10, interferon-γ (IFN-γ), and tumor necrosis factor-α (TNF-α) in serum were determined using the swine enzyme-linked immunosorbent assay kit (Abcam, United Kingdom) according to the manufacturer’s instructions.

### Gene Sequencing of 16S rRNA of Gut Microbes

The microbial genomic DNA was extracted from intestinal content samples using Fast DNA SPIN extraction kits (MP Biomedicals, Santa Ana, CA, United States) according to the manufacturer’s instructions. The quantity and quality of extracted DNAs were measured using a NanoDrop ND-1000 spectrophotometer (Thermo Fisher Scientific, Waltham, MA, United States) and agarose gel electrophoresis, respectively. In this experiment, the microbes’ V3–V4 hypervariable regions of 16S rRNA gene, with a length of approximately 500 bp, were used for sequencing. The primer sequences were 338F 5′-ACTCCTACGGGAGGCAGCA-3′ and 806R 5′-GGACTACHVGGGTWTCTAAT-3′. The polymerase chain reaction (PCR) condition was as follows: initial denaturation at 98°C for 2 min, followed by 25 cycles consisting of denaturation at 98°C for 15 s, annealing at 55°C for 30 s, and extension at 72°C for 30 s, with a final extension of 5 min at 72°C. The PCR amplicons were purified with Agencourt AMPure Beads (Beckman Coulter, Indianapolis, IN, United States) and quantified using the PicoGreen dsDNA Assay Kit (Invitrogen, Carlsbad, CA, United States). After that, the individual quantification steps were pooled in equal amounts paired-end 2 bp × 250 bp sequencing was performed using the Illumina MiSeq platform with MiSeq Reagent Kit v3 at Shanghai Personal Biotechnology, Co., Ltd. (Shanghai, China).

### Analysis of Biodiversity

Illumina MiSeq sequences were analyzed by Quantitative Insights Into Microbial Ecology, v1.8.0 (QIIME) (Caporaso et al., 2010) for taxonomic analysis at phylum and genus levels. The operational taxonomy units (OTUs) were defined as sequences clustered with a similarity cutoff of 97% using UCLUST algorithm (Edgar et al., 2011). Alpha diversity indices were determined using Mothur (Schloss et al., 2009).

### Statistical Analysis

The data of immune indices were presented as mean ± standard deviation and analyzed using one-way analysis of variance (one-way ANOVA), followed by LSD multiple comparison tests by SPSS software (IBM SPSS Statistics 20 for windows). The value of *p* < 0.05 was considered statistically significant. Canonical correspondence analysis (CCA) was performed using Canoco for Windows 4.5 between the first 20 predominant genera and the immune indices in each group. Correlation analysis between key communities and immune indices were determined based on Pearson’s rank correlation coefficient.

## Results

### Performance and Occurrence of Diarrhea

The performance and occurrence of diarrhea are shown in Table 2. Piglets sensitized with soybeans had lower average daily gain and feed conversion ratio (*p* < 0.05) compared with the control group. However, supplementation with FOS increased the daily weight gain reaching values similar to the control group. Furthermore, occurrence of diarrhea decreased in the FOS group (8.75%), compared with the allergy group (22.50%).

**Table 2:** Effects of FOS on performance^a^, diarrhea^b^, total serum IgG and IgE levels, glycinin, and β-conglycinin-specific IgG antibody OD units and cytokine concentrations in serum in soybean allergy protein-sensitized and control piglets.

Item	Control	FOS	Allergy	R-MSE	*p*-Value
Average daily gain (g/d)	234.51 ± 30.47^a^	236.08 ± 46.19^a^	180.45 ± 31.40^b^	16.43	0.034
Average daily feed intake (g/d)	501.51 ± 12.46	525.71 ± 56.46	481.44 ± 28.09	17.30	0.084
F/G	2.20 ± 0.23^b^	2.26 ± 0.20^b^	2.73 ± 0.49^a^	0.15	0.026
Occurrence of diarrhea (%)	0	8.75	22.5		
Total serum IgG (mg/mL)	16.95 ± 2.80^b^	20.58 ± 2.42^a^	21.34 ± 1.84^a^	2.08	0.014
Total serum IgE (μg/mL)	110.58 ± 12.59^b^	154.54 ± 7.06^a^	158.45 ± 19.37^a^	11.39	0.006
Glycinin specific IgG OD units	0.656 ± 0.027^b^	0.631 ± 0.062^b^	0.727 ± 0.047^a^	0.033	0.042
β-Conglycinin specific IgG OD units	0 .609 ± 0.057^b^	0.525 ± 0.049^b^	0.702 ± 0.085^a^	0.037	0.005
Cytokine concentrations in serum (pg/mL)					
IFN-γ	566.03 ± 41.12^a^	529.60 ± 30.51^a^	453.78 ± 24.12^b^	20.01	0.01
IL-4	292.95 ± 10.76^b^	278.01 ± 11.32^b^	349.78 ± 15.32^a^	11.19	<0.01
IL-10	106.27 ± 31.70^b^	153.36 ± 44.66^b^	260.09 ± 82.55^a^	25.58	<0.01
IL-6	61.75 ± 10.81	62.75 ± 7.88	66.00 ± 6.67	42.06	0.451
TNF-α	44.81 ± 16.61	50.33 ± 7.85	53.83 ± 16.94	7.81	0.342


*^a^Each value is the mean of data from five piglets (average initial BW of 6.81 ± 0.58 kg) per group.*

*^*b*^Diarrhea (%) = (Number of diarrhea pigs × Days of diarrhea)/(Total number of pigs × Total number of text days) × 100%.*

*Dissimilar letters represent significant difference among different groups (*p* < 0.05).*

### Total Serum IgG and IgE and Specific IgG Levels

To explore the effects of FOS on soybean-induced allergy in sensitized pigs, the total serum IgG, IgE, glycinin-specific IgG, and β-conglycinin-specific IgG levels were determined (Table 2). The total serum IgG and IgE were significantly higher in the allergy group and the FOS group compared with the control group (*p* < 0.05). Glycinin-specific IgG and β-conglycinin-specific IgG were significantly higher in the allergy group compared with the control group (*p* < 0.05); however, no differences were observed between the FOS group and the control group.

### Serum Cytokine Levels

To determine whether oral FOS administration altered Th1/Th2 cytokine levels in response to soybean stimulation, serum IL-4,IL-10, and IFN-γ levels were measured. The results showed that IFN-γ level was significantly higher (*p* < 0.05), while IL-4 and IL-10 levels were significantly lower in the control and FOS groups compared with the allergy group (*p* < 0.05). There were no differences for IL-6 and TNF-α among different groups (*p* > 0.05) (Table 2).

### Analysis of Gut Microbes

A total of 1,282,521 sequences were obtained from the piglets gut microbes, with an average of 35,626 sequences per sample (30, 230-60, 640 sequences). The bacterial diversity of the intestinal content samples in different groups is presented in Table 3. The Chao 1 and Ace indices in cecum were significantly higher than in other intestinal segments (*p* < 0.05) (Table 3A). The M-jejunum had lower Shannon and Simpson than ileum and cecum (*p* < 0.05). As shown in Table 3B, Chao1, ACE, and Shannon of ileum in the FOS and allergy groups were significantly higher compared with the control group (*p* < 0.05). The *S*hannon indices ranged from 6.777 to 8.193 in three groups, respectively. There were no statistically significant differences in diversity estimators between M-jejunum and P-jejunum (*p* > 0.05).

**Table 3:** The bacterial diversity of the intestinal content samples based on Miseq of the 16S rRNA gene.

(A) The comparison among four intestinal segments-ignored groups.				
	M-Jejunum	P-Jejunum	Ileum	Cecum

Chao1	1279.08 ± 197.92^b^	1419.47 ± 144.14^b^	1425.42 ± 300.40^b^	1721.15 ± 286.64^a^
Ace	1287.90 ± 181.72^b^	1442.22 ± 158.27^b^	1439.30 ± 307.76^b^	1754.34 ± 296.62^a^
Shannon	6.918 ± 0.436^b^	7.327 ± 0.520^ab^	7.614 ± 0.439^a^	7.826 ± 0.348^a^
Simpson	0.9556 ± 0.019^b^	0.966 ± 0.013^ab^	0.975 ± 0.006^a^	0.970 ± 0.012^a^

**(B) The comparison among three groups in each intestinal segment.**			

	**Control**	**0.6%FOS**		**Allergy**

**M-jejunum**			
Chao1	1200.42 ± 297.38	1370.15 ± 150.19		1266.67 ± 154.11
Ace	1218.92 ± 262.77	1378.12 ± 138.80		1266.67 ± 154.11
Shannon	6.777 ± 0.434	7.000 ± 0.701		6.977 ± 0.188
Simpson	0.954 ± 0.019	0.957 ± 0.007		0.956 ± 0.032
**P-Jejunum**			
Chao1	1463.44 ± 109.93	1418.74 ± 92.46		1376.22 ± 238.26
Ace	1490.42 ± 103.46	1433.72 ± 100.94		1402.52 ± 270.82
Shannon	7.21 ± 0.632	7.16 ± 0.651		7.61 ± 0.271
Simpson	0.961 ± 0.013	0.974 ± 0.003		0.964 ± 0.019
**Ileum**			
Chao1	1247.22 ± 144.30^b^	1506.36 ± 114.69^a^		1716.35 ± 52.28^a^
Ace	1260 ± 153.69^b^	1508.21 ± 114.66^a^		1744.70 ± 79.92^a^
Shannon	7.123 ± 0.065^b^	7.89 ± 0.29^a^		7.83 ± 0.372^a^
Simpson	0.972 ± 0.006	0.976 ± 0.011		0.974 ± 0.006
**Cecum**			
Chao1	1732.15 ± 275.23	1701.28 ± 249.15		1730.02 ± 435.82
Ace	1809.87 ± 281.42	1739.30 ± 314.97		1713.85 ± 407.56
Shannon	7.467 ± 0.137^c^	8.193 ± 0.138^a^		7.817 ± 0.227^b^
Simpson	0.960 ± 0.015	0.979 ± 0.001		0.972 ± 0.006


*Dissimilar letters represent significant difference among different groups (*p* < 0.05).*

**FIGURE 1:**
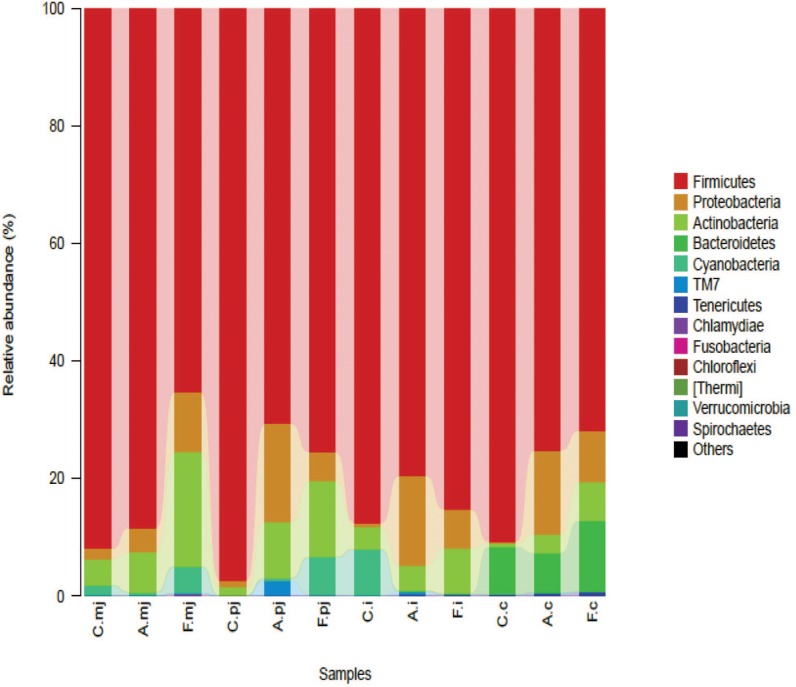
Bacterial compositions in the different groups and intestinal segments at the phylum level.

At t*h*e phylum level, *Firmicutes* was the most-dominant phylum among all intestinal segments, followed by *Proteobacteria*, *Actinobacteria*, *Cyanobacteria*, and *Bacteroidetes* (Figure 1). For P-jejunum and cecum, the abundance of *Firmicutes* in the allergy group was significantly lower compared with the control group (*p* < 0.05) (Table 4). In contrast, the ratio of *Proteobacteria* was significantly higher in the allergy group than the control group in all intestinal segments. Compared to the control and allergy groups, *Proteobacteria* showed a dynamic pattern in the FOS group: a 10.18% of increase at M-jejunum, a 4.92% of decrease at P-jejunum, and a 6.61% of decrease at ileum, and an 8.74% of increase at cecum. *Cyanobacteria* in ileum was significantly lower in the allergy group than in the control group. *Actinobacteria* in M-jejunum was significantly higher in the FOS group than the allergy and control groups (*p* < 0.05). The abundance of *Bacteroidetes* increased more than 7–10-fold at cecum than other segments.

**Table 4:** Profiles of gut microbes in all intestinal segments and groups at the rank of phylum according to taxon-based analysis.

	Firmicutes (%)	Proteobacteria (%)	Cyanobacteria (%)	Actinobacteria (%)	Bacteroidetes (%)
C-mj	91.99 ± 1.34^a^	1.71 ± 2.66^b^	1.72 ± 1.66	4.52 ± 1.78^b^	0.01 ± 0.01
A-mj	88.45 ± 8.21^a^	4.14 ± 1.89^a^	0.50 ± 0.55	6.87 ± 9.51^b^	0.01 ± 0.01
F-mj	65.21 ± 3.65^b^	10.18 ± 5.47^a^	4.44 ± 3.71	19.64 ± 8.44^a^	0.03 ± 0.003
C-pj	97.52 ± 2.47^a^	1.06 ± 1.52^b^	0.01 ± 0.01^b^	1.42 ± 1.06	0.00 ± 0.00
A-pj	70.68 ± 14.11^b^	16.67 ± 7.25^a^	0.42 ± 0.70^b^	9.69 ± 15.49	0.04 ± 0.05
F-pj	75.55 ± 12.92^b^	4.92 ± 3.13^b^	6.45 ± 11.10^a^	12.91 ± 10.88	0.05 ± 0.06
C-i	87.61 ± 0.51	0.59 ± 0.11^b^	7.91 ± 0.51^a^	3.87 ± 0.48	0.01 ± 0.00
A-i	79.58 ± 7.39	15.19 ± 5.16^a^	0.06 ± 0.05^b^	4.37 ± 5.98	0.15 ± 0.16
F-i	85.28 ± 12.01	6.61 ± 7.50^b^	0.06 ± 0.07^b^	7.73 ± 10.84	0.13 ± 0.23
C-c	90.82 ± 6.42^a^	0.28 ± 0.14^b^	0.02 ± 0.02	0.60 ± 0.44	8.09 ± 6.62
A-c	75.23 ± 7.22^b^	14.35 ± 0.19^a^	0.05 ± 0.07	3.15 ± 1.98	6.80 ± 4.93
F-c	71.92 ± 5.76^b^	8.74 ± 2.40^a^	0.03 ± 0.01	6.57 ± 3.39	12.13 ± 7.01


*C: control group; A: allergy group; F: 0.6% FOS; mj: M-jejunum; pj: P-jejunum; i: ileum; c: cecum. Dissimilar letters represent significant difference in the same segment among different groups (*p* < 0.05).*

**FIGURE 2:**
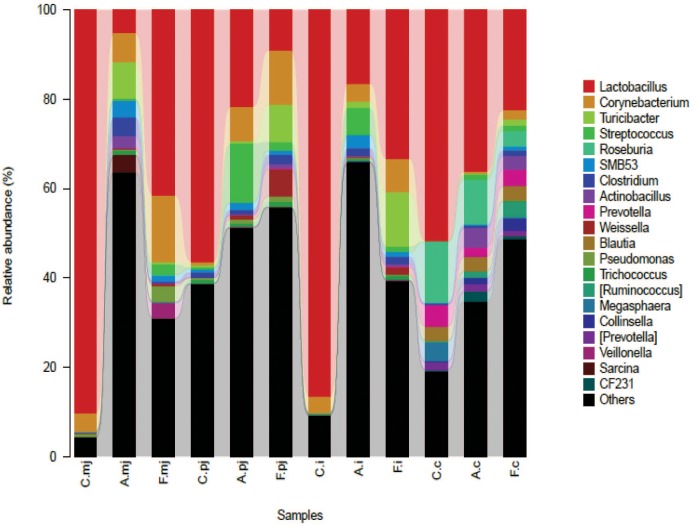
Bacterial compositions in the different groups and intestinal segments at the genus level.

At the genus level, a total of 183 genera were identified from all samples. The abundance of *Lactobacillus* made it the most-dominant bacterium in all genus (Figure 2) and was significantly lower in the allergy group at M-jejunum, P-jejunum, and ileum than the control group (*p* < 0.05) (Table 5). The abundance of *Lactobacillus* at cecum was 51.73% in the control group and 36.21% in the allergy group. However, there was no significant difference between these two groups (*p* > 0.05). Compared to the allergy group, *Lactobacillus* showed a dynamic pattern in the FOS group with a 41.48% of increase at M-jejunum, an 8.97% of decrease at P-jejunum, a 33.16% of increase at ileum, and a 22.51% of decrease at cecum. *Bifidobacteria* is a minor community in intestinal microbes, which was enriched in the FOS group in M-jejunum. The abundance of *Streptococcus* was significantly higher in the allergy group at P-jejunum and ileum (*p* < 0.05). Moreover, *Clostridiaceae* was significantly higher in the allergy group at M-jejunum and P-jejunum compared to the control group (*p* < 0.05), whereas it was significantly decreased in the FOS group compared with the allergy group. In addition, *Turicibacter*, *Peptostreptococcaceae* also changed as shown in Table 5.

**Table 5:** Dominance of gut microbes in all intestinal segments and groups at the rank of genus according to taxon-based analysis.

	*Lactobacillus* (%)	*Clostridiaceae* (%)	*Turicibacter* (%)	*Streptococcus* (%)	*Peptostreptococcaceae* (%)	*Bifidobacteria* (%)
C-mj	90.39 ± 2.34^a^	0.478 ± 0.36^b^	0.02 ± 0.002^b^	0.05 ± 0.01	0.04 ± 0.06	0.14 ± 0.06^b^
A-mj	5.06 ± 2.15^c^	53.88 ± 7.49^a^	8.07 ± 6.67^a^	0.52 ± 0.09	4.99 ± 2.20	0.05 ± 0.07^b^
F-mj	41.48 ± 17.6^b^	7.46 ± 8.89^b^	0.461 ± 0.36^b^	2.30 ± 1.80	0.72 ± 0.80	3.59 ± 4.68^a^
C-pj	56.39 ± 6.52^a^	0.33 ± 0.07^c^	0.032 ± 0.03^b^	0.36 ± 0.62^b^	0.03 ± 0.02^b^	0.05 ± 0.07
A-pj	21.64 ± 15.28^b^	36.10 ± 11.53^a^	1.39 ± 0.69^b^	13.27 ± 14.63^a^	8.85 ± 1.70^a^	0.33 ± 0.54
F-pj	8.97 ± 6.05^b^	20.53 ± 15.11^b^	12.33 ± 7.64^a^	1.81 ± 2.57^b^	8.08 ± 3.99^a^	0.41 ± 0.13
C-i	86.65 ± 0.32^a^	28.08 ± 4.51	0.59 ± 0.66^b^	0.05 ± 0.02^b^	6.45 ± 6.21^a^	0.05 ± 0.00
A-i	16.42 ± 9.19^b^	23.50 ± 17.21	0.59 ± 0.94^b^	6.00 ± 3.92^a^	2.38 ± 2.64^b^	0.08 ± 0.09
F-i	33.16 ± 26.76^b^	34.15 ± 1.85	8.47 ± 8.22^a^	1.07 ± 1.34^b^	8.08 ± 6.32^a^	0.07 ± 0.10
C-c	51.73 ± 9.33^a^	0.96 ± 0.75	0.08 ± 0.11	0.01 ± 0.02	0.21 ± 0.26	0.00 ± 0.00
A-c	36.21 ± 17.1^ab^	1.73 ± 0.54	0.23 ± 0.08	1.09 ± 0.66	0.68 ± 0.31	0.00 ± 0.01
F-c	22.51 ± 15.3^b^	4.95 ± 3.86	1.32 ± 1.15	1.12 ± 0.48	1.28 ± 0.91	0.05 ± 0.05


*C: control group; A: allergy group; F: 0.6% FOS; mj: M-jejunum; pj: P-jejunum; i: ileum; c: cecum. Dissimilar letters represent significant difference in the same segment among different groups (*p* < 0.05).*

### Correlation Analysis Between Immune Indices and Gut Microbes

**FIGURE 3:**
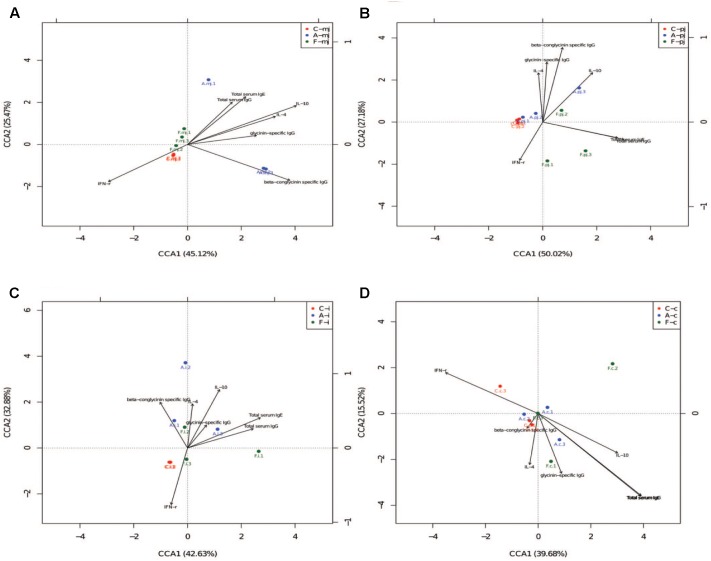
The CCA of all intestinal segments between immune index and first 20 species of bacteria at genus level. **(A)** M-jejunum; **(B)** P-jejunum; **(C)** ileum **(D)** cecum. “∙” indicates the first 20 species of bacteria genus of each group. Arrow indicates immune index. The closer the pendulum is to the arrow, the greater the positive correlation between bacteria genus and the immune index; the pendulum is farther away from the arrow, which indicates negative correlation between bacteria genus and the immune index. If the angle between the arrows is acute, immune indexes were a positive correlation; the converse indicated negative correlation.

Canonical correspondence analysis reflects the relationship between microbes and environmental factors (immune indices). As shown in Figure 3, we found that specific IgG, IL-4, IL-10, and total serum IgG and IgE were correlated negatively to IFN-γ. Microbes in the allergy group were correlated positively to specific IgG, IL-4, and IL-10 and were correlated negatively to IFN-γ. Microbes in the control group were correlated positively to IFN-γ. Microbes in the FOS group were correlated positively to total serum IgG and IgE.

We also performed an association analysis based on the Pearson’s rank correlation coefficient using different taxa by pooling these three groups together. At the phylum level (Table 6), *Firmicutes* was correlated positively to the level of β-conglycinin-specific IgG in M-jejunum and was correlated negatively to the level of IL-10 in P-jejunum and glycinin-specific IgG in ileum (*p* < 0.05). *Proteobacteria* was correlated positively to the expression of IL-4 in P-jejunum and cecum and the level of IL-10 in P-jejunum, ileum, and cecum (*p* < 0.05); it was correlated negatively to the expression of IFN-γ in P-jejunum, ileum, and cecum (*p* < 0.05). *Actinobacteria* and *Cyanobacteria* were correlated negatively to the expression of β-conglycinin-specific IgG in M-jejunum (*p* < 0.05). *Cyanobacteria* was correlated negatively to the level of total serum IgG, IgE, and IL-10 in ileum (*p* < 0.05).

**Table 6:** Phyla correlated to the key communities of intestinal flora and Pearson’s correlation between phyla and immune index.

Genus	Total serum IgG Pearson’s correlation	β-Conglycinin specific IgG Pearson’s correlation	Glycinin-specific IgG Pearson’s correlation	Total serum IgE Pearson’s correlation	IL-4 Pearson’s correlation	IFN-γ Pearson’s correlation	IL-10 Pearson’s correlation
**M-jejunum**							
Firmicutes	–0.628	0.729*	0.517	–0.566	0.482	–0.999	0.144
Proteobacteria	0.485	–0.237	–0.273	0.410	–0.367	–0.236	0.100
Actinobacteria	0.519	–0.691*	–0.498	0.481	–0.372	0.139	–0.152
Bacteroidetes	0.04	–0.285	–0.609	–0.033	–0.558	0.022	–0.185
Cyanobacteria	0.347	–0.689*	–0.217	0.330	–0.340	0.402	–0.328
**P-Jejunum**							
Firmicutes	–0.595	–0.455	–0.242	–0.575	–0.198	0.537	–0.720*
Proteobacteria	0.248	0.574	0.301	0.389	0.676*	–0.832*	0.713*
Actinobacteria	0.584	0.183	0.095	0.459	–0.043	–0.006	0.341
Bacteroidetes	0.348	–0.262	–0.431	0.283	–0.095	–0.112	0.073
Cyanobacteria	0.031	–0.177	–0.228	0.022	–0.464	–0.131	0.025
**Ileum**							
Firmicutes	–0.37	–0.254	–0.822*	–0.47	–0.523	0.479	–0.642
Proteobacteria	0.339	0.408	0.514	0.460	0.555	–0.828*	0.83*
Actinobacteria	0.487	–0.170	0.377	0.517	0.052	0.084	0.120
Bacteroidetes	0.655	0.065	0.620	0.700*	0.361	–0.246	0.494
Cyanobacteria	–0.813*	–0.117	–0.118	–0.898*	–0.226	0.607	–0.683*
**Cecum**							
Firmicutes	–0.559	0.229	0.482	–0.455	0.34	–0.073	–0.011
Proteobacteria	0.542	0.491	0.637	0.705*	0.701*	–0.824*	0.893*
Actinobacteria	0.537	–0.152	0.251	0.598	–0.082	–0.259	0.343
Bacteroidetes	–0.063	0.203	0.210	–0.072	–0.139	–0.326	0.144
Cyanobacteria	0.320	0.523	0.702*	0.234	0.331	–0.321	0.533


*^∗^The correlation is significant at a level of 0.05.*

At the genus level (Table 7), *Lactobacillus* was correlated negatively to total serum IgG, IgE, and IL-10 and was correlated positively to IFN-γin all intestinal segments. *Clostridiaceae*, *Peptostreptococcaceae*, and *Enterobacteriaceae* were correlated positively to the expression of β-conglycinin-specific IgG, IL-4, and IL-10 (*p* < 0.05) and were correlated negatively to the level of IFN-γ in M-jejunum (*p* < 0.05). *Clostridiaceae* and *Peptostreptococcaceae* were correlated positively to the level of IL-10 (*p* < 0.05) and were correlated negatively to IFN-γ in ileum. *Enterobacteriaceae* was correlated positively to β-conglycinin-specific IgG, glycinin-specific IgG, IL-4, and IL-10 (*p* < 0.05) and correlated negatively to IFN-γ (*p* < 0.05) in cecum. *Streptococcus* was correlated positively to glycinin and β-conglycinin-specific IgG and IL-10 in P-jejunum (*p* < 0.05). Total serum IgG and IgE were correlated positively to *Peptostreptococcaceae*, *Turicibacter*, *Streptococcus*, *Clostridiales*, and *Clostridium* in all intestinal segments.

**Table 7:** Genera correlated to the key communities of intestinal flora and Pearson’s correlation between genus and immune index.

Phyla	Total serum IgG Pearson’s correlation	β-Conglycinin specific IgG Pearson’s correlation	Glycinin-specific IgG Pearson’s correlation	Total serum IgE Pearson’s correlation	IL-4 Pearson’s correlation	IFN-γ Pearson’s correlation	IL-10 Pearson’s correlation
**M-jejunum**							
Lactobacillus	–0.726*	–0.436	–0.454	–0.873*	–0.639*	0.742*	–0.863*
Clostridiaceae	0.403	0.779*	0.702*	0.538	0.867*	–0.779*	0.921*
Turicibacter	0.249	0.879*	0.522	0.349	0.614	–0.553	0.716*
Streptococcus	0.299	–0.352	–0.325	0.301	–0.495	–0.143	0.041
Clostridiales	0.550	–0.594	–0.085	0.577	–0.209	0.134	–0.119
Peptostreptococcaceae	0.374	0.836*	0.624	0.499	0.764*	–0.691*	0.844*
Bifidobacteria	0.528	–0.558	–0.049	0.563	–0.187	0.127	–0.114
Enterobacteriaceae	0.298	0.893*	0.608	0.409	0.777*	–0.729*	0.871*
Clostridium	0.452	0.672*	0.425	0.568	0.627	–0.605	0.686*
**P-Jejunum**							
Lactobacillus	–0.805	–0.067	0.058	–0.825*	0.056	0.396	–0.491
Clostridiaceae	–0.187	–0.625	–0.750*	–0.126	–0.452	0.249	–0.512
Turicibacter	0.609	–0.593	–0.278	0.611	–0.277	0.228	–0.297
Streptococcus	0.343	0.775*	0.742*	0.350	0.519	–0.485	0.793*
Clostridiales	0.086	–0.378	–0.138	0.235	–0.223	–0.001	–0.267
Peptostreptococcaceae	0.126	–0.504	–0.168	0.221	–0.341	0.071	–0.337
Bifidobacteria	0.642	0.223	0.439	0.574	0.070	–0.222	0.510
Enterobacteriaceae	0.052	0.438	0.129	0.215	0.624	–0.635	0.473
Clostridium	0.343	–0.548	–0.392	0.411	–0.285	0.130	–0.337
**Ileum**							
Lactobacillus	–0.676*	–0.325	–0.395	–0.812*	–0.421	0.726*	–0.793*
Clostridiaceae	0.476	0.547	0.426	0.624	0.471	–0.769*	0.808*
Turicibacter	0.683*	–0.612*	–0.42	0.648*	–0.375	0.220	–0.170
Streptococcus	0.109	0.569	0.226	0.285	0.609	–0.743*	0.612
Clostridiales	0.613	0.195	–0.031	0.664*	0.148	–0.593	0.625
Peptostreptococcaceae	0.584	0.187	0.098	0.632	0.175	–0.629	0.680*
Bifidobacteria	0.426	0.182	0.625	0.468	0.265	–0.084	0.350
Enterobacteriaceae	0.160	0.071	0.138	0.282	0.320	–0.667*	0.495
Clostridium	0.667	0.187	0.290	0.790*	0.269	–0.671*	0.701*
**Cecum**							
Lactobacillus	–0.677*	0.118	0.236	–0.695*	0.131	0.369	–0.44
Clostridiaceae	0.329	–0.277	–0.186	0.305	–0.421	–0.211	0.091
Turicibacter	0.545	–0.538	–0.078	0.576	–0.252	–0.053	–0.016
Streptococcus	0.666*	–0.158	0.209	0.761*	0.270	–0.579	0.557
Clostridiales	0.724*	0.227	0.352	0.773*	0.142	–0.617	0.727
Peptostreptococcaceae	0.733*	–0.468	–0.013	0.747*	0.028	–0.224	0.134
Bifidobacteria	0.496	–0.491	–0.044	0.532	–0.328	–0.016	0.001
Enterobacteriaceae	0.321	0.722*	0.728*	0.440	0.805*	–0.847*	0.962*
Clostridium	0.341	–0.219	–0.048	0.321	–0.310	–0.311	0.189


*^∗^The correlation is significant at a level of 0.05.*

## Discussion

Soybean antigen protein (particularly glycinin and β-conglycinin) often impairs the performance and immune function of swine (Sun et al., 2008, 2009), such as average daily gain and feed conversion, and causes diarrhea, which is similar to the findings in our study. We also found that 0.6% FOS enhanced the performance of swine and reduced the occurrence of diarrhea, which is consistent with previous studies (Oli et al., 1998; Estrada et al., 2001; Nakamura et al., 2010; Apper et al., 2016).

Previous studies have shown that soybean antigen-induced anaphylaxis mainly includes IgE-mediated type I allergic reaction and IgG-mediated type IV allergic reaction (Sun et al., 2008). Glycinin and β-conglycinin play an important role in the majority of soybean-induced anaphylaxis. The levels of glycinin and β-conglycinin-specific IgG were increased after the soybean-induced anaphylaxis (Burks et al., 1988). Similarly, a previous study also showed that soybean extracts increased soybean antigen-specific IgG level in the serum of infants with allergies, which were confirmed in our study. Furthermore, we found that glycinin and β-conglycinin-specific IgG levels decreased in the FOS group; so, we speculated that FOS may suppress the specific IgG antibody by modulating gut microbes and then alleviate soybean-induced anaphylaxis. In this study, we used biotinylated antipig IgG due to the lack of antipig IgE antibody, which has limited the unequivocal identification of antigen-specific IgE in sensitized pigs.

In addition, total serum IgG and IgE antibody levels were increased in the allergy group, which were similar to the findings from other studies (Friesen et al., 1993; Sun et al., 2009; Yang et al., 2018). Previous studies have shown that FOS can enhance immunity by increasing total serum IgG in swine (Apper et al., 2016; Wang et al., 2017). Similarly, they were increased in FOS group compared with control group.

The analysis of serum cytokines showed that piglets with FOS supplementation had higher interferon-γ level, but lower levels of IL-4 and IL-10 compared with the allergy group. The IFN-γ is a symbolic cytokine of Th1 cells. Inflammation factors of IL-4 and IL-10 are secreted by Th2 cells, which stimulate the proliferation of B lymphocytes and generate IgE and IgG antibodies (Yamada et al., 2012; Canani and Costanzo, 2013; Aldaghri et al., 2014), which play a key role in allergic inflammation (Prescott and Hogan, 2006). Vos et al. (2007) showed that GOS/FOS can reduce the Th2 response and increase the activation of Th1 pathway when Balb/c mice were sensitized with ovalbumin. These results suggest that the antihypersensitivity effect might be mediated by reversing the ratio of Th1/Th2 cells in the allergic subjects. In conclusion, suppressive effects of FOS on soybean antigen-induced anaphylaxis may be regulated by inflammatory factors and the balance between Th1 and Th2 immunity.

Gut microbes play a crucial role in the establishment of tolerance to food proteins. In this study, the microorganisms in the sensitized group changed significantly, indicating the damages to the intestinal microenvironment in piglets sensitized by soybeans. The phyla of *Firmicutes* and the genus of *Lactobacillus* were decreased in the allergy group. A previous study found a similar result that soybean antigen, such as β-conglycinin, can inhibit the proliferation of *Lactobacillus* and *Bifidobacteria* (Hao et al., 2010). *Lactobacillus* protects the host against potential pathogen invasions (Martín et al., 2013), promotes the mucosal immune response (Erickson and Hubbard, 2000), modulates the cytokine expression, and stimulates the production of Tr1 and Th3 cytokines (Smits et al., 2005; Frossard et al., 2007). It was reported that oral FOS affects intestinal microbes, especially the abundance of *Lactobacillus* and *Bifidobacteria* (Ohta et al., 2002; Langlands et al., 2004). Both *Lactobacilli* and *Bifidobacteria* have been shown to improve the increased gut permeability during the exposure to food allergens (Rautava et al., 2005). In the FOS group, *Lactobacillus* was higher in M-jejunum and ileum, whereas *Bifidobacteria* also increased in M-jejunum. A recent research has shown that a chronic enrichment of *Proteobacteria* in the gut, including *Enterobacteriaceae*, can represent an imbalanced/unstable microbial community structure or a state of disease of the host (Shin et al., 2015). *Proteobacteria* are a major phylum of gram-negative bacteria, which can increase lipopolysaccharide endotoxin in the blood, decrease the number of intestinal barrier cells, and increase intestinal permeability, leading to chronic inflammatory response (Cani et al., 2007). In our study, *Proteobacteria* was significantly higher in the allergy group in all intestinal tracts and decreased in the FOS group at P-jejunum and ileum. Therefore, consumption of FOS might modulate the intestinal microbes, improve the gut permeability, and decrease the allergy reaction. We also observed *Turicibacter* enrichment in the FOS group at P-jejunum and ileum. Similarly, supplementation of stachyose and barley malt can increase the relative abundance of *Turicibacter* (Zhong et al., 2015; Liu et al., 2018). Some pathogenic bacteria, including *Streptococcus*, can increase the levels of IL-6, IL-8, and IL-10 (Van den Bogert et al., 2014). Our study showed that *Streptococcus* was increased significantly in the allergy group and decreased in the FOS group at P-jejunum and ileum.

Studies show that microbes have modulatory effects on regulatory T-cells, which provides mutual benefits and regulations between the host immune system and microbes (Hrncir et al., 2008; Round and Mazmanian, 2010; Wei et al., 2010). We found that *Lactobacillus* was correlated negatively to the expression of IL-10 and total serum IgE and was correlated positively to the expression of IFN-γ. Previous studies reported that *Lactobacillus* suppressed T-cell proliferation and Th2 cytokines secretion from allergic objects (Von der Weid et al., 2001; Lin et al., 2006), inhibited IgE responses and systemic anaphylaxis in a murine model of food allergy (Shida et al., 2002), and enhanced IFN-γ production (Pohjavuori et al., 2004). *Proteobacteria* promotes colonic inflammation (Maharshak et al., 2013) and induces IL-10 production (Devkota et al., 2012). We found that *Proteobacteria* and genus of *Enterobacteriaceae* were correlated positively to IL-4 and IL-10 and were correlated negatively to IFN-γ. Van et al. (2010) showed that FOS can promote immune function in swine by enhancing total serum IgG and IgE. The genus of *Peptostreptococcaceae*, *Turicibacter*, *Streptococcus*, *Clostridiales*, and *Clostridium* were correlated positively to total serum IgG and IgE.

## Conclusion

Our study demonstrates that oral supplementation of FOS can effectively relieve anaphylaxis induced by the soybean antigen in piglets. This protective mechanism is associated with the suppression of specific IgG and inflammatory cytokine release as well as the change in gut microbes. Improvement of intestinal microbes is mainly indicated by the increase of *Lactobacillus* and *Bifidobacteria* in M-jejunum and the decrease in *Proteobacteria* in P-jejunum and ileum. In addition, FOS can increase the total serum IgG and IgE to enhance the immune function. Our results provide a new and effective solution for the alleviation of allergic symptoms in livestock and humans and provide new hope for the development of prebiotics for the prevention and treatment of allergic reactions.

## Author Contributions

MC and YZ designed and wrote this manuscript. XZ was involved in modifying the manuscript. GQ supervised the writing of this manuscript. All authors read and approved the final manuscript.

## Conflict of Interest Statement

The authors declare that the research was conducted in the absence of any commercial or financial relationships that could be construed as a potential conflict of interest.
